# Fear-learning is altered in a mouse neuropathic pain model

**DOI:** 10.3389/fpain.2025.1648374

**Published:** 2025-08-29

**Authors:** Neda Assareh, Eddy E. Sokolaj, Saima Sadia, Kristen E. Anderson, Caitlin Frith, Olivia B. Walls, Vanessa A. Mitchell, Christopher W. Vaughan, Bryony L. Winters

**Affiliations:** ^1^ Pain Management Research Institute, Kolling Institute, University of Sydney at Royal North Shore Hospital, Sydney, NSW, Australia; ^2^ Charles Perkins Centre, Sydney Pharmacy School, University of Sydney, Sydney, NSW, Australia

**Keywords:** neuropathic pain, fear, fear-conditioning, fear-learning, freezing, anxiety, allodynia

## Abstract

**Background:**

While chronic neuropathic pain is characterised by abnormal pain signs, such as allodynia, highly disabling co-morbidities, such as anxiety and depression, have a major impact. It is thought that these co-morbidities arise from learning maladaptations related to inappropriate associations between pain and stimulus/environmental cues. However, the impact of animal neuropathic pain models on the interactions between fear-learning, pain and anxiety are poorly understood, particularly during early stages prior to establishment of anxiety.

**Methods:**

We examined the impact of fear-conditioning on fear, anxiety-like behaviours and cold/mechanical allodynia in the mouse sciatic nerve chronic constriction injury (CCI) model of neuropathic pain, at an early post-injury time point.

**Results:**

At 2 weeks post-surgery, CCI and sham operated mice displayed similar acquisition of fear-like freezing responses to a paired audio-tone/footshock fear-conditioning paradigm. On the following day, CCI mice displayed greater freezing than sham mice in response to the same context and subsequent tone presentations. While CCI and sham mice display similar anxiety-like behaviour in the light-dark box and open field, these were increased by fear-conditioning in CCI but not mice. Finally, CCI but not sham surgery produced cold and mechanical allodynia, however, these were unaffected by fear-conditioning.

**Conclusions:**

These findings indicate that a neuropathic pain model enhances learned context/cue evoked fear behaviours at an early stage following nerve-injury. Furthermore, fear-conditioning enhances anxiety-like behaviour, before such behaviour is normally developed. Thus, fear-conditioning induces exaggerated fear-learning which triggers enhanced fear and anxiety, even during early stages of chronic neuropathic pain.

## 1 Introduction

Chronic neuropathic pain is a highly prevalent and debilitating pain syndrome which is caused by damage to the somatosensory nervous system through nerve trauma, diseases such as diabetes, and medications such as chemotherapy drugs (1). Chronic neuropathic pain sufferers experience an abnormal pain syndrome which commonly includes spontaneous pain, cold and mechanical hypersensitivity (allodynia), all of which are largely refractory to current medications (2, 3). Compounding this abnormal pain syndrome, neuropathic pain is associated with psychological disturbances including anxiety, distress and depression (4, 5).

Like humans, nerve injury in rodents produces mechanical and cold allodynia (6, 7). Importantly, allodynia in animal models has a rapid onset, mechanistically transitioning from an acute to a chronic pain phase within 3–5 days following nerve injury (8, 9). More recent studies have shown that animal neuropathic pain models also induce anxiety and depression-like behaviours, becoming apparent at approximately 4-weeks following nerve injury (10–14). Thus, anxiety-like co-morbidities have relatively delayed expression compared to the abnormal chronic pain in these animal models. It is unclear whether this temporal difference between the onset of chronic pain and its co-morbidities is fixed or can be altered by additional stressors.

While fear-learning is an adaptation that normally triggers protective behaviours, avoidance models have proposed that chronic pain states are associated with a vicious cycle of altered fear-learning which leads to anxiety, depression and other co-morbidities (15–17). Indeed, clinical studies have suggested that fear-learning is altered in various chronic pain syndromes (18–22). Fear-conditioning has long been used in animal studies to explore the mechanisms underlying learned fear behaviours associated with various anxiety disorders (23). However, there are relatively few studies on the impact of fear-conditioning on fear and anxiety-like behaviours in animal neuropathic pain models (13, 24, 25). Furthermore, the relative timing of nerve-injury to fear-conditioning varies substantially between these studies. We therefore focused on whether fear-conditioning alters the relative expression of pain and associated co-morbidities at an early time-point post-nerve-injury, where allodynia has a chronic phenotype, but prior to the enhancement of anxiety-like behaviour.

## 2 Methods

All experiments in this study were carried out on 9–12-week-old male C57BL/6 mice obtained from Australia Bio Resources (Moss Vale, Australia) and approved by the University of Sydney Animal Ethics Committees (protocols RESP-18-321, 2022-2229). All data are reported in compliance with the ARRIVE guidelines and those of the “NH&MRC Code of Practice for the Care and Use of Animals in Research in Australia”. Mice were housed in groups of 4 littermates in environmentally enriched ventilated cages which were maintained at 22°C–23°C and 65%–75% humidity, with a 12:12 h light: dark cycle. Animals had *ad libitum* access to food and water throughout all stages of the study.

### 2.1 Neuropathic pain model

Chronic constriction injury (CCI) of the left sciatic nerve was used to model neuropathic pain (6, 7). For surgery, mice were anesthetized (2% isoflurane in saturated O_2_) and placed on a heated mat. In CCI-operated mice, the left common sciatic nerve was exposed, and two 6-0 chromic gut ligatures were placed 2 mm apart around the common sciatic nerve. The ligatures were lightly tightened until a brief foot twitch was observed, taking care not to compromise nerve blood flow. In sham-operated mice, the sciatic nerve was exposed but left untouched. In both surgery groups, the muscle over the nerve was closed with 6-0 silk, and the skin incision closed with tissue glue. After recovery from anesthesia, mice were housed in individual cages for 1–2 days to ensure healing of the incision site, then returned to their group home cages. Mice were monitored every 1–3 days until the day of the experiment.

### 2.2 Pain and behavioural testing

Chronic pain was manually assessed by measuring cold and mechanical allodynia in real-time by the experimenter. These are reproducible chronic pain signs which are stable over several weeks following CCI surgery (7). Mice were placed in an elevated Perspex chamber with a wire mesh floor and left to acclimatize for 30–60 min. Cold allodynia was assessed by counting the number of pain-like responses (rapid flinching, shaking or licking of the hindpaw) to evaporative cooling induced application of acetone (20 µl) on the plantar surface of the operated hind paw over a 30-s period. Mechanical allodynia was assessed by applying a series of mechanical forces with graded von Frey filaments (0.2–6.84 g; North Coast Medical, San Jose, CA) to the plantar surface of the operated hind paw. Testing started with the 1.0 g hair, then proceeded to a higher or lower forces depending upon the lack or presence of a pain-like response, respectively, for a total of 5 times. The mechanical paw-withdrawal threshold (PWT) was calculated using the simplified up-down method (26).

Anxiety-like behaviour was assessed with the light-dark box and open-field tests. The light-dark box test was conducted in a double chamber (height 30 cm) with a light open-top side (28 × 28 cm, white walls and floor, under 65 lx illumination) and dark closed-lid side (14 × 26 cm, matt black walls, floor and ceiling), separated by an opening (70 × 75 cm), and with an overhead camera on the light side to record activity. Mice were individually placed in the light zone, approximately two thirds away from the entrance to the dark zone and activity recorded for 5 min. The open field test was conducted in an open top chamber (50 × 50 × 50 cm white floor and walls) with overhead illumination (65 lx), and an overhead camera to record activity. Mice were individually placed in the middle of the open-field chamber and activity was recorded for 15 min. After testing, mice were immediately returned to their home cage.

### 2.3 Fear-conditioning

Fear-conditioning (FC) was performed in footshock chambers with a stainless-steel grid floor through which a scrambled electric current was applied (18 × 18 × 16 cm, Med Associates, Fairfax, USA). Each footshock chamber was inside an adjacent outer sound isolated cabinet (80 × 55 × 45 cm) that was ventilated (exhaust fan, noise = 70 dB inside the footshock chamber), and under moderate illumination (6 lx warm white light). Each footshock chamber had a speaker to transmit an audio-tone (5 kHz, 80 dB) and an overhead camera record activity.

**Figure 1:**
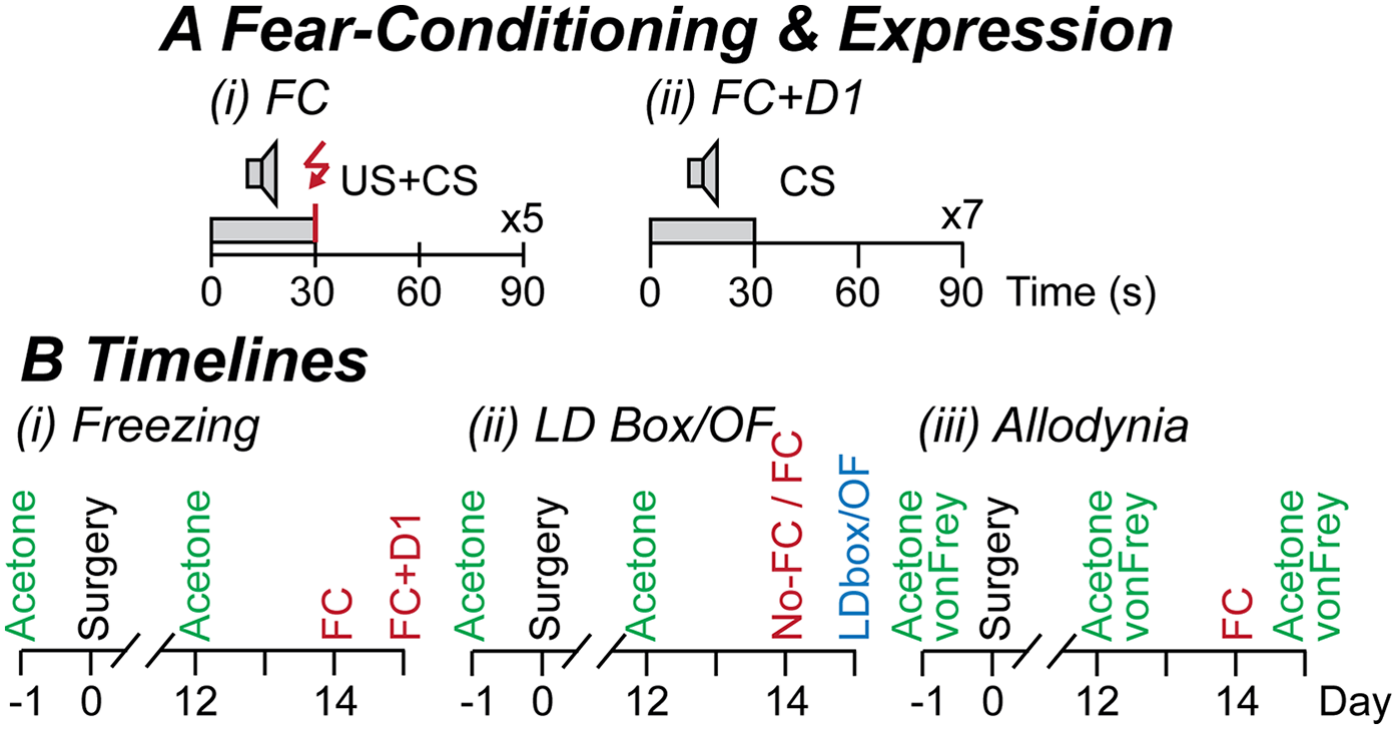
Experimental protocols. **(A)** The fear-conditioning (FC) protocol consisted of (i) 5 repetitions of a 30 s conditioned stimulus (CS, audio-tone at 5 kHz, 80 dB) which co-terminated with a 1 s unconditioned stimulus (US, footshock at 0.9 mA), and in some experiments, then (ii) assessment of fear-learning on the following day (FC + D1): 7 repetitions of the 30 s CS; at an interval of 90 s on both days. **(B)** Timelines of separate experimental cohorts in which mice underwent 1-week acclimatization, pain testing (acetone and von Frey testing only in cohort 3), sham or CCI surgery on the following day (day 0), then pain testing at 12-days post-surgery. For (Bi) cohort 1 freezing experiments: mice underwent fear-conditioning at 14-days post-surgery, then assessment of fear-learning on the following day; (Bii) cohort 2 light-dark (LD) box and open-field (OF) experiments: mice underwent fear-conditioning (FC) or no fear-conditioning (no-FC) at 14-days post-surgery, then LD box or OF testing on the following day; (Biii) cohort 3 allodynia experiments: mice underwent fear-conditioning at 14-days post-surgery, then acetone and von Frey testing on the following day.

For fear-conditioning, mice were placed in the footshock chambers and acclimatized for 4 min. A conditioned stimulus (CS, audio-tone, 80 dB, 30 s duration), which co-terminated with an aversive unconditioned stimulus (US, electrical footshock, 0.9 mA, 1 s duration), was presented 5 times at 90 s intervals (Figure 1A). Mice were left in the chamber for 1 min following stimulus presentation, then returned to their home cages. In experiments examining the expression of fear-learning, mice were placed in the footshock chambers on the following day and after 2 min the CS-alone was presented 7 times at 90 s intervals (Figure 1A). All testing devices were cleaned with 80% ethanol and 1% acetic acid before and after use.

### 2.4 Experimental protocol

Mice were acclimatized to their housing, experimenter handling, and the allodynia testing chamber over a 1-week period. They were then assigned to either CCI-operated, or sham-operated groups before any procedures were performed (two of each per experimental group of four mice). Mice underwent testing for cold allodynia (cohorts 1–3) and mechanical allodynia (only cohort 3) on the day prior to, then at 12 days following sham- or CCI-surgery. The impact of fear-conditioning on freezing behaviour, anxiety-like behaviours, and allodynia was then examined in separate cohorts because of potential carry-over effects between the different types of behavioural tests, as follows, then immediately euthanized by CO_2_ asphyxiation and cervical dislocation:

• Cohort 1: fear-like freezing behaviour (Figure 1B). All mice underwent a US + CS fear-conditioning session at 14-days post-surgery, then on the following day were exposed to CS presentations in the same chamber (context) used for fear-conditioning.
• Cohort 2a/b: anxiety-like behaviours (Figure 1B). Separate mice underwent either fear-conditioning or were placed inside the chamber for an equal duration and did not undergo fear-conditioning at 14-days post-surgery. On the following day, separate groups then underwent testing in the light-dark box or open-field.
• Cohort 3: neuropathic pain (Figure 1B). All mice underwent fear-conditioning at 14-days post-surgery. On the following day, they then underwent cold then mechanical allodynia testing.

### 2.5 Analysis and statistics

Surgery groups were not blinded during the experiments as neuropathic pain signs were readily detected by experimenters. However, all groups were then blinded until data had been analyzed, collated and quantified. No data was excluded except for one experiment (cohort 1, CCI mice) in which the video file was corrupted, rendering it unreadable.

Freezing, a standard fear-like behaviour in rodents (27), was automatically assessed using ezTrack (28). To do this, motion was quantified as the number of frame-by-frame pixel fluctuations above a fixed noise threshold. The presence of freezing was then scored when motion was below a fixed cut-off threshold for at least 1 s These thresholds were set before analyzing all the video files, with (i) the noise threshold set at the 99.99 percentile of random pixel fluctuations in empty chambers, and (ii) the cut-off level set to at a value which gave optimal correlation between manually observed freezing and that detected by ezTrack in sample blinded videos. Freezing was averaged over 30 s epochs before, during and after CS, or US + CS presentations on days 0 and 1, respectively. Data for day 0 US + CS presentations were analyzed individually, and for day 1 CS1 was analyzed individually, and CS2-3, 4-5 and 6-7 were averaged in pairs. Light-dark box and open-field behaviour were automatically quantified using ANY-Maze (Stoelting Co.). Light-dark box behaviour was quantified as the number of entries into and percentage time spent within the light zone over a 5-min period, and open-field behaviour was quantified as the total distance travelled and percentage time in the central zone over a 15-min period (ANY-Maze).

Data was analyzed using two-way ANOVAs, or two-way repeated measures (RM) ANOVAs with main effects of surgical group (between-subject: sham, CCI) and time (within-subject, for cohorts 1 and 3), or treatment (between-subject: fear-conditioning, no-fear-conditioning, for cohort 2) where appropriate (Prism, GraphPad Software). Data satisfied the Brown-Forsythe test for homogeneity of variance, and the Greenhouse-Geisser correction was applied for RM ANOVAs when Mauchly's test for sphericity was violated. All data are presented as the mean ± S.E.M and classified as significantly different when *p* < 0.05. When ANOVA main effects were significant, posthoc comparisons were made using Dunnett's, Sidak's or Fisher's LSD multiple comparison tests where appropriate, and values are shown as *p* > 0.05, or *p* < 0.05, 0.01, 0.001, 0.0001.

## 3 Results

### 3.1 Effect of CCI nerve injury on the fear-conditioning acquisition and recall

**Figure 2:**
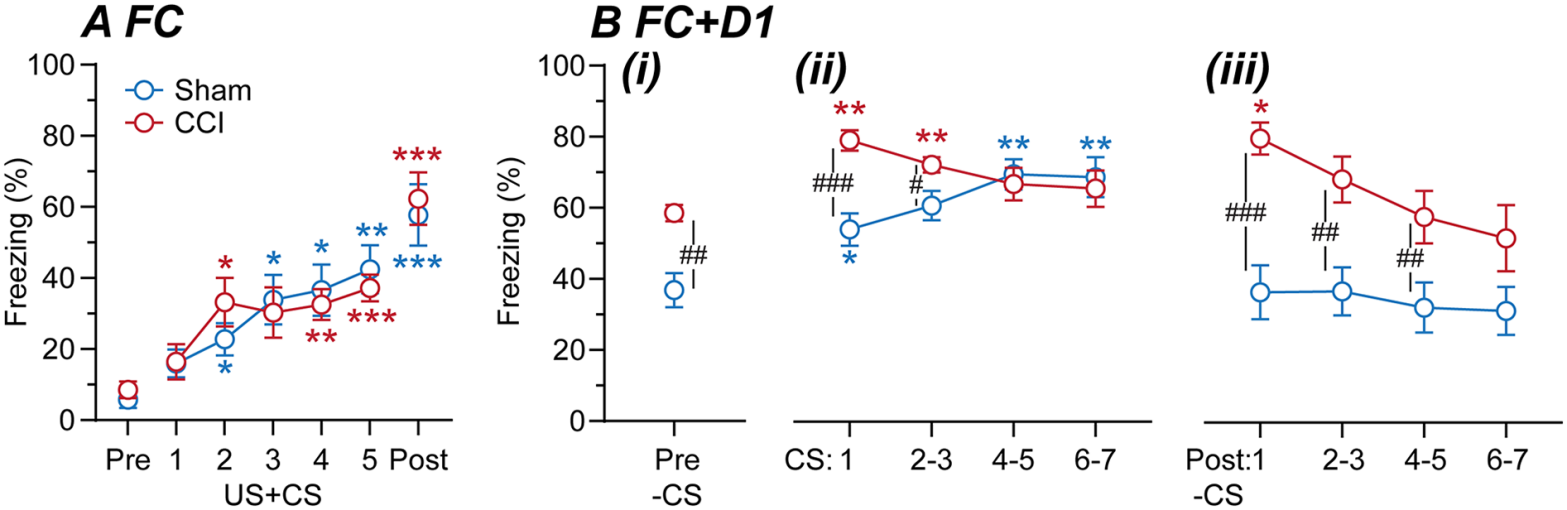
Fear-learning in sham vs. CCI mice. Time plots of the percentage time sham and CCI mice spent freezing during **(A)** fear-conditioning at 14-days post-surgery and **(B)** assessment of fear recall on the following day (*n* = 10, 9 per group). In **(A)**, freezing is shown for time points before fear-conditioning (Pre), during the 5× footshock + tone presentations (US + CS1-5), and following the final US + CS presentation (Post). In **(B)**, freezing is shown for (i) before (Pre), (ii) during and (iii) following 7× tone presentations (CS1, 2/3, 4/5, 6/7). Dunnett's multiple comparisons: *, **, *** denote *p* < 0.05, 0.01, 0.001 for each time point vs. the **(A)** pre-US + CS, or **(B)** pre-CS time points in sham and CCI-mice. Sidak's multiple comparisons: #, ##, ### denote *p* < 0.05, 0.01, 0.001 for sham v CCI at each time point.

In the first cohort of mice, we examined whether CCI nerve injury alters fear-learning. At 14 days post-surgery, sham and CCI-operated animals underwent fear-conditioning, with five audio-tones (CS) paired with footshocks (US) (*n* = 10, 9; sham, CCI mice). There was a significant main effect of time but not surgical group, nor was there an interaction between these factors for the percentage time freezing [F (4.2, 71.1) =  30, *p* <  0.0001, F(1, 17) = 0.02, *p* > 0.05, F(4.2, 71) = 0.8, *p* >  0.05, 2-way RM ANOVA]. There was a progressive increase in freezing during paired US + CS presentations in both sham and CCI mice and following the final US + CS presentation, when compared to the pre-US + CS level (Figure 2A). There was no significant difference in freezing between CCI and sham mice at any of these time points (Figure 2A).

On the day following fear-conditioning, these mice were placed in the same chamber/environmental context and after 2-min were repeatedly exposed to the audio-tone alone (CS). There were significant main effects of time and surgery, and an interaction between these factors for the percentage time freezing [F (3.6, 64) = 9.9, *p* < 0.0001, F (1, 18) = 15, *p* < 0.01, F (3.6, 64) = 5.1, *p* < 0.01, 2-way RM ANOVA]. Freezing was greater in CCI than sham mice when initially placed in the chamber, prior to first CS presentation (Figure 2B). Following this, there was a significant increase in freezing during most of the CS presentations compared to the pre-CS time point in both sham and CCI mice (Figure 2B). However, freezing during the early CS presentations was greater in CCI than sham mice (Figure 2B). In addition, freezing was greater in CCI than sham mice following most CS presentations (Figure 2B). Interestingly, freezing remained elevated above the pre-CS level in CCI but not sham mice following the CS1 presentation (Figure 2B). These observations indicate that CCI nerve injury increases learned fear responses to both context and cue-presentations in that environmental context.

### 3.2 Impact of CCI nerve injury and fear-conditioning on anxiety-like behaviours

**Figure 3:**
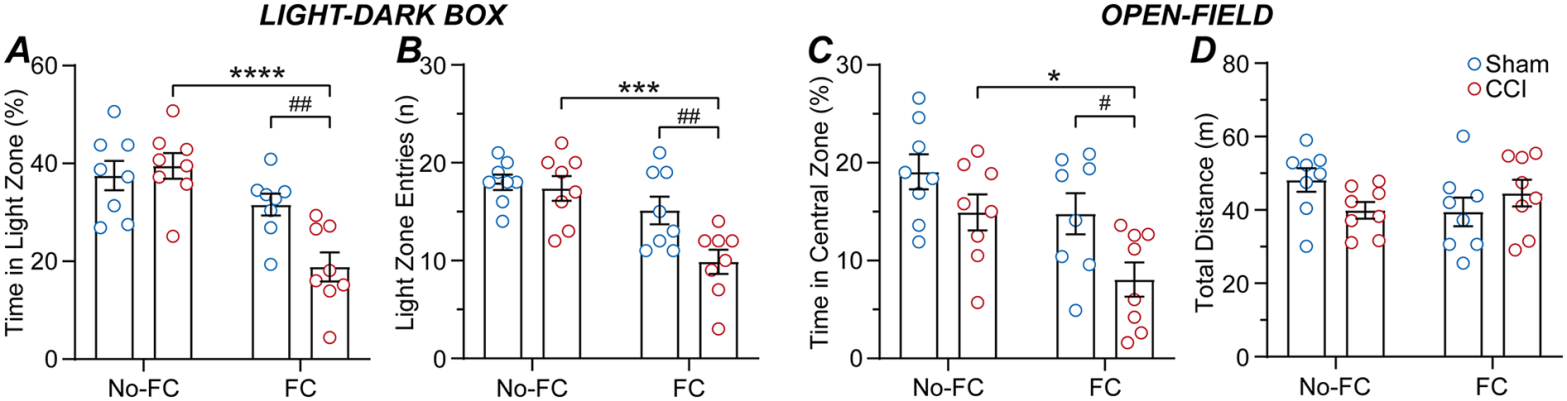
CCI nerve injury alters the impact of fear-conditioning on anxiety-like behaviours. Bar/scatter plots of **(A)** the time spent in the light-zone and **(B)** number of entries into the light-zone of the light-dark box, and **(C)** time spent in the central-zone and **(D)** total distance travelled within the open-field, in sham and CCI operated mice (*n* = 8 per group). Sham and CCI mice underwent fear-conditioning (FC) or just placement inside the same chamber without fear-conditioning (no-FC) at 14-days post-surgery, then light-dark box or open-field testing at 15-days post-surgery. Fisher's LSD multiple *post hoc* comparisons: *, ***, **** denote *p* < 0.05, 0.001, 0.0001 for no-FC v FC, and #, ## denote *p* < 0.05, 0.01 for sham v CCI.

In the second cohort of mice, we examined the impact of the CCI neuropathic pain model and subsequent fear-conditioning on light-dark box and open-field behaviour, in two separate groups of mice. At 14-days post-surgery, sham and CCI mice underwent either fear-conditioning or no fear-conditioning, and on the following day, testing in either the light-dark box or open-field (*n* = 8 per sham and CCI mouse group). In the light-dark box, there was a significant main effect of fear-conditioning but not surgery, and an interaction between these factors for the percentage time spent within the light zone [F (1, 28) = 24, *p* < 0.0001, F (1, 28) = 3.9, *p* > 0.05, F (1, 28) = 7.3, *p* < 0.05, 2-way ANOVA]. In addition, there were significant main effects of fear-conditioning and surgery, and an interaction between these factors for the number of entries into the light zone [F (1, 28) = 19, *p* < 0.001, F (1, 28) = 6.0, *p* < 0.05, F (1, 28) = 4.3, *p* < 0.05, 2-way ANOVA]. In the animals that did not undergo fear-conditioning, there was no significant difference between sham and CCI mice for the number of entries into, or the percentage time spent within the light zone (Figures 3A,B). By contrast, CCI mice that underwent fear-conditioning spent significantly less time within and fewer entries into the light zone than their sham counterparts (Figures 3A,B). In addition, CCI but not sham mice that underwent fear-conditioning made fewer entries into and spent less time within the light zone than those that did not undergo fear-conditioning (Figures 3A,B).

For the open-field, there were significant main effects of fear-conditioning and surgery, but no interaction between these factors for the time in the central zone [F (1, 28) = 8.4, *p* < 0.01, F (1, 28) = 8.8, *p* < 0.01, F (1, 28) = 0.5, *p* > 0.05, 2-way ANOVA]. There was no significant difference in the time spent in the central zone between sham and CCI mice which did not undergo fear-conditioning, (Figure 3C). However, CCI mice that underwent fear-conditioning spent less time within the central zone than their sham counterparts (Figure 3C). Thus, CCI but not sham mice that underwent fear-conditioning spent less time in the central zone than those that did not undergo fear-conditioning (Figure 3C). There was no significant main effect of fear-conditioning or surgery, or an interaction between these factors for the total distance travelled [F (1, 28) = 0.2, *p* > 0.05, F (1, 28) = 0.4, *p* > 0.05, F (1, 28) = 3.9, *p* > 0.05, 2-way ANOVA] (Figure 3D). Together, these observations indicate that suggests that while CCI nerve injury does not increase anxiety-like behaviours, these behaviours are enhanced by fear-conditioning in CCI but not sham mice.

### 3.3 Effect of fear conditioning on allodynia

**Figure 4:**
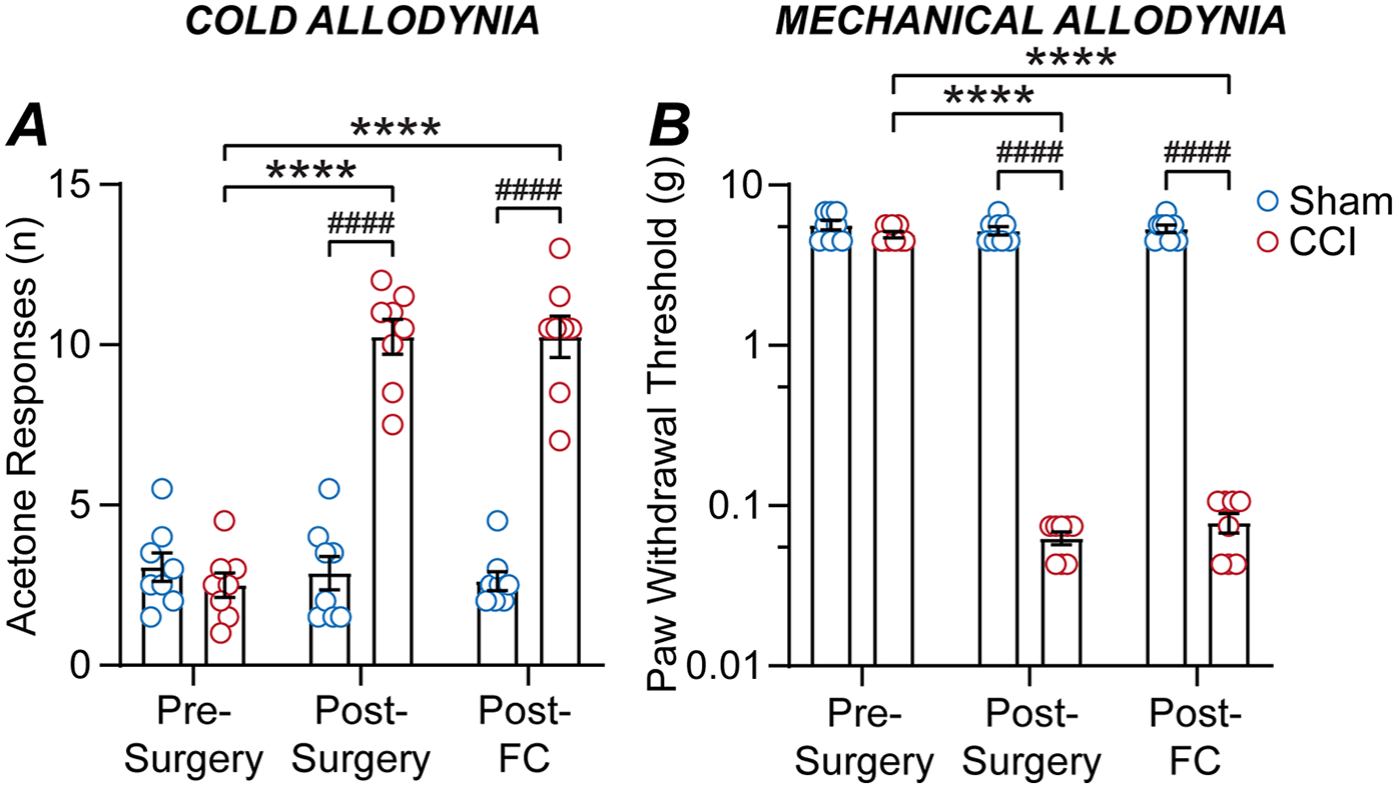
CCI nerve injury but not fear-conditioning alters allodynia. Bar/scatter plots of **(A)** acetone responses and **(B)** mechanical paw-withdrawal thresholds in sham and CCI mice. Data is shown for allodynia prior to surgery (pre-surgery), at 12-days post-CCI/sham surgery (post-surgery), then on the day following fear-conditioning at 14-days post-surgery (post-FC). Sidak's multiple comparisons: **** denote *p* < 0.0001 for pre- vs. post-surgery and post-FC, and #### denotes *p* < 0.0001 for sham v CCI.

In the final cohort of mice, we examined the impact of the CCI neuropathic pain model and subsequent fear-conditioning on allodynia. Mice underwent both acetone and von Frey hair testing for cold and mechanical allodynia prior to sham/CCI surgery, at 12-days following surgery, then at 14-days following surgery on the day following fear-conditioning (*n* = 8, 8 sham, CCI mice). For acetone responses, there were significant main effects of time and surgery, and an interaction between these factors [F (1.3, 19) = 77, *p* < 0.0001; F (1, 14) = 75, *p* < 0.0001; F (1.3, 19) = 90, *p* < 0.0001, two-way RM ANOVA]. For mechanical paw-withdrawal threshold, there were significant main effects of time and surgery, plus and a significant interaction between these factors [F (1.5, 21) = 83, *p* < 0.0001; F (1, 14) = 251, *p* < 0.0001; F (1.5, 21) = 61, *p* < 0.0001, two-way RM ANOVA]. There was no difference in the basal number of acetone responses, or mechanical paw-withdrawal thresholds between the sham and CCI groups prior to surgery (Figures 4A,B).

As observed previously, there was a significant increase in the number of acetone responses and a decrease in the mechanical paw-withdrawal threshold for CCI but not sham mice at 12-days post-surgery compared to pre-surgery levels (Figures 4A,B). However, there was no difference in acetone responses on the day following fear-conditioning compared to the post-surgery level, for both sham and CCI mice (Figure 4A). Likewise, there was no difference in mechanical paw-withdrawal threshold on the day following fear-conditioning compared to the post-surgery level, for both sham and CCI mice (Figure 4B). Thus, while CCI nerve injury induces cold and mechanical allodynia, fear-conditioning does not alter cold or mechanical sensitivity in either CCI or sham operated mice.

## 4 Discussion

In the present study, it has been demonstrated that the sciatic nerve CCI model of neuropathic pain alters learned fear recall in mice. While nerve-injury did not alter fear responses during the acquisition phase, it enhanced the fear recall responses to the environmental context and audio cues within that context on the following day. Nerve-injury also enhanced pain-like responses but had no effect on anxiety-like behaviour. However, fear-conditioning enhanced anxiety-like behaviour in nerve-injured mice but had no effect on their pain-like responses. Together, these findings suggest that this neuropathic pain model primes systems which control anxiety and fear, thereby facilitating stressor-induced enhancement of these behaviours.

The impact of a neuropathic pain state on fear-learning was examined by comparing fear conditioning in mice at 2-weeks following CCI sciatic nerve injury compared control sham surgery. It was observed that CCI and control sham mice displayed similar progressive increases in freezing during paired audio-tone/footshock (US + CS) presentations, as observed previously (13). On the day following fear acquisition, reintroduction into the same context induced greater freezing in CCI-injured compared to control sham mice. While this hasn't been examined previously in nerve-injury models, it is consistent with the enhancement of contextual fear-learning previously observed in a rat diabetic model of neuropathic pain (29, 30). Subsequent audio-tone presentations in the same environmental context also induced greater freezing in CCI compared to sham mice, particularly during earlier presentations. This differs from prior studies in which nerve-injury had no effect on subsequent fear-conditioning induced fear recall (13, 24). This difference might be due to the prior use of less intense fear-conditioning paradigms and examination of cue-induced freezing in a novel context (13, 24). Another difference is that fear-conditioning in one of these studies was induced at 2-days after nerve-injury (24). It is important to emphasize that this is a time point at which pain has not yet transitioned from an acute to chronic phase (9). Finally, it should be noted that unlike sham mice, freezing remained elevated following each audio-tone cue cessation in CCI mice. Together, these findings indicate that the CCI nerve-injury neuropathic pain model enhances fear responses to context and cues, when presented within that context, and blunts the ability to terminate fear responses following cessation of a learned cue. It remains to be determined how the individual roles of cue and context in fear-learning are altered in this neuropathic pain model.

It was observed that there was no difference in basal anxiety-like behaviour between CCI and control sham mice, as assessed with the light-dark box and open-field tests. This was observed as decreased entries and time within the light-zone of the light-dark box and decreased time within the central zone of an open-field, as reported in prior studies at 1–2 weeks following nerve-injury (31–37). This differs from other studies where neuropathic pain models produce an increase in these anxiety-like behaviours at 4–8 weeks post-injury (10–13, 38). This difference is consistent with the proposition that enhanced anxiety-like behaviours are not observed until approximately 4-weeks after nerve injury (39). Interestingly, we observed that fear-conditioning produced increased anxiety-like behaviour in both the light-dark box and open-field in CCI, but not control sham mice. Together, these data suggest that while nerve injured animals did not display greater anxiety-like behaviour than sham controls, an increase in this behaviour was triggered by fear-conditioning. These findings are consistent with the demonstration that, in addition to freezing behaviour, fear-conditioning can lead to non-associative sensitization (40, 41). Thus, fear-anxiety control systems appear to be primed at 2-weeks following nerve injury, prior to the expression of any overt anxiety-like behaviour, and this can be unmasked by a stressor such as fear-conditioning. These changes could be mediated by multiple brain regions and mechanisms, which need to be explored in future studies. These include regions in which nerve-injury is known to alter various neurotransmitter systems and plasticity such as the amygdala, locus coeruleus, nucleus accumbens and cortex that have a role in the complex (13, 17, 42, 43).

Finally, it was observed that CCI nerve-injury produced cold and mechanical allodynia at 2-weeks post-surgery, as we have observed previously (7, 44). Interestingly, cold and mechanical allodynia were unaffected by fear-conditioning in the CCI nerve-injured mice, nor was cold/mechanical sensitivity affected by fear-conditioning in sham operated mice. Thus, unlike freezing and anxiety-like behaviour in nerve-injured mice, fear-conditioning had no effect on cold and mechanical allodynia, both of which are abnormal pain sensations normally associated with this neuropathic pain state (3). It would, however, be important to compare male and female mice in future studies, to identify potential differences in the impact of fear-conditioning on pain, fear, and anxiety-like behaviour in neuropathic pain models.

Overall, the present study has demonstrated that learned fear responses are altered by a nerve-injury model of neuropathic pain, at least in the context in which those fear memories are induced. While nerve-injury induced allodynia, it did not affect anxiety-like behaviour at an early post-injury time point. Furthermore, fear-conditioning enhanced anxiety-like behaviour but had no effect on allodynia at this early phase of neuropathic pain. Thus, our findings demonstrate that an early imbalance in fear-acquisition and recall is induced by nerve-injury which can then trigger enhanced fear and anxiety, a common comorbidity associated with chronic neuropathic pain.

## Data Availability

The raw data supporting the conclusions of this article will be made available by the authors, without undue reservation.
